# Estimating the Impact of Novel Digital Therapeutics in Type 2 Diabetes and Hypertension: Health Economic Analysis

**DOI:** 10.2196/15814

**Published:** 2019-10-09

**Authors:** Robert J Nordyke, Kevin Appelbaum, Mark A Berman

**Affiliations:** 1 Beta6 Consulting Group, LLC Topanga, CA United States; 2 Better Therapeutics, LLC San Francisco, CA United States

**Keywords:** digital therapeutics, behavioral intervention, economic evaluation, diabetes, hypertension

## Abstract

**Background:**

Behavioral interventions can meaningfully improve cardiometabolic conditions. Digital therapeutics (DTxs) delivering these interventions may provide benefits comparable to pharmacologic therapies, displacing medications for some patients.

**Objective:**

Our objective was to estimate the economic impact of a digital behavioral intervention in type 2 diabetes mellitus (T2DM) and hypertension (HTN) and estimate the impact of clinical inertia on deprescribing medications.

**Methods:**

Decision analytic models estimated health resource savings and cost effectiveness from a US commercial payer perspective. A 3-year time horizon was most relevant to the intervention and payer. Effectiveness of the DTx in improving clinical outcomes was based on cohort studies and published literature. Health resource utilization (HRU), health state utilities, and costs were drawn from the literature with costs adjusted to 2018 dollars. Future costs and quality-adjusted life years (QALYs) were discounted at 3%. Sensitivity analyses assessed uncertainty.

**Results:**

Average HRU savings ranged from $97 to $145 per patient per month, with higher potential benefits in T2DM. Cost-effectiveness acceptability analyses using a willingness-to-pay of $50,000/QALY indicated that the intervention would be cost effective at total 3-year program costs of $6468 and $6620 for T2DM and HTN, respectively. Sensitivity analyses showed that reduced medication costs are a primary driver of potential HRU savings, and the results were robust within values tested. A resistance to deprescribe medications when a patient’s clinical outcomes improve can substantially reduce the estimated economic benefits. Our models rely on estimates of clinical effectiveness drawn from limited cohort studies with DTxs and cannot account for other disease management programs that may be implemented. Performance of DTxs in real-world settings is required to further validate their economic benefits.

**Conclusions:**

The DTxs studied may provide substantial cost savings, in part by reducing the use of conventional medications. Clinical inertia may limit the full cost savings of DTxs.

## Introduction

Intensive behavioral and lifestyle interventions have been shown to meaningfully improve clinical outcomes in patients with various cardiometabolic conditions, providing potential for substantial reduction in medication and other resource use. For example, structured, comprehensive lifestyle change programs improve glycemic control in type 2 diabetes mellitus (T2DM), with a substantial number of patients seeing benefits that are comparable or greater than those achieved by pharmacotherapy [1-5]. Behavioral interventions have also demonstrated the ability to control and in some cases achieve normal blood pressure in patients with hypertension [6,7]. Behavioral interventions have thus established themselves as essential complements to pharmacologic therapy, which is reflected in current treatment guidelines [8-10]. Behavioral interventions also have potential as alternatives to conventional pharmacologic therapy for some patients. For example, participants in the LookAHEAD trial maintained significant improvements over a standard education program in body weight, hemoglobin A_1c_ (HbA_1c_), systolic blood pressure (SBP), and low-density lipoprotein cholesterol at 4 years, enabling a meaningful proportion of patients to eliminate or reduce antidiabetic pharmacotherapy [1,11].

More recently, mobile software apps have been shown to provide effective platforms to deliver behavioral interventions to patients with cardiometabolic and addictive conditions. The ease of implementation and use of software to treat disease (referred to as a digital therapeutic [DTx]) may help overcome the difficulty of health care systems to deploy intensive behavioral interventions at the large scale needed to improve population outcomes. Moreover, the mobile nature of these apps allows patients to engage with the intervention program several times per day, which may drive improved outcomes over conventional delivery [12]. In cohort studies of T2DM patients, DTxs have demonstrated improvements in clinical outcomes. A mobile medical app that delivered intensive behavioral therapy paired with support from a remote multidisciplinary care team demonstrated mean improvements of HbA_1c_ of 0.8% over a 3-month study period, with improvements up to 1.3% for patients with higher levels of engagement [12]. A similar DTx designed for hypertensive patients demonstrated the ability to reduce blood pressure in a 3-month study with mean reductions in SBP of 11.5 mm Hg and reductions of 17.6 mm Hg among participants with stage 2 hypertension (HTN) [13]. These early results suggest that DTxs may provide clinical benefits comparable to pharmacologic therapy and, in some patients, may help reduce or eliminate the need for medications.

In current clinical practice, however, there is often a delay in deprescribing medications even when the need to do so has been established. This widespread phenomenon, known as clinical inertia, contributes to polypharmacy, which leads to adverse drug reactions, unnecessary costs, and worsened quality of life for patients [14,15]. Clinical inertia, if not addressed, could also lessen the economic benefits realized when a digital therapeutic is put into practice.

The economic benefits of conventionally delivered lifestyle interventions have been demonstrated based on randomized clinical trials [16,17]. However, at this point in their development and introduction into clinical practice, there are few formal evaluations of the potential economic benefits of mobile-platform DTxs and none, that we are aware of, that incorporate measures of clinical inertia. A recent systematic review of a variety of digital health tools showed them to be highly cost effective, although only one study in a Spanish treatment setting evaluated a mobile app comparable to DTxs considered here [18,19]. Thus, our objective in this analysis was to explore the potential economic benefits of DTxs for the treatment of distinct, high-cost cardiometabolic diseases. We developed economic models for the use of DTxs in T2DM and HTN addressing clinical inertia from the perspective of US commercial payers.

## Methods

### Model Setting

To understand the relative impact of DTxs in two different cardiometabolic disease states, we created a common framework to estimate the impact of implementing a DTx in distinct patient populations with primary diagnoses of T2DM or HTN. This common framework was implemented in Excel-based decision tree models created from the US payer perspective to best reflect real-world data on medication costs, accurately reflect attrition from the DTx intervention, and account for the currently limited data on DTx effectiveness. A common model framework also facilitates transparent comparisons across disease states modeled. Participants were assumed to enroll in the DTx program at the beginning of year 1 of a 3-year intervention with attrition occurring throughout the program. A 3-year time horizon was chosen as most appropriate for US commercial health plans implementing a behavioral intervention because these plans experience significant annual enrollee turnover and prior studies show that the impact of behavioral intervention can wane over the course of several years [11]. These factors tend to make the longer term benefits to the initiating plan less meaningful financially. We referred to the Consolidated Health Economic Evaluation Reporting Standards (CHEERS) guidelines to improve reporting of this economic analysis [20].

**Figure 1:**
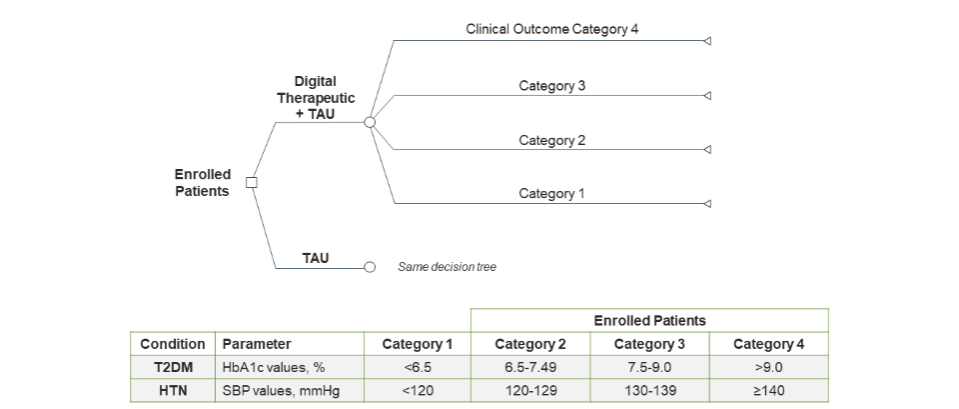
Model structure.

The models were based on the observation that biomarker elevation correlates with both clinical events and health resource utilization (HRU) [21-23]. Each model compared two cohorts (Figure 1): DTx + treatment as usual (TAU) and TAU alone. At this point in their development, economic evaluations of digital health interventions are commonly conducted using a treatment-as-usual comparator [18]. Clinical outcomes were classified into one of four categories chosen to leverage costs reported in the literature and where possible align with current clinical guidelines [8,9,21]. For T2DM, these were defined by HbA_1c_ values of <6.5 (category 1), 6.5 to 7.49% (category 2), 7.5% to 9.0% (category 3), and >9% (category 4). For HTN, these were defined in terms of SBP: <120 mm Hg (category 1), 120 to 129 mm Hg (category 2), 130 to 139 mm Hg (category 3), and >140 mm Hg (category 4). Enrollees were assumed to have active disease with the primary diagnosis corresponding to each model; no patients with already optimized biomarkers were enrolled. Since enrollees were not naïve to conventional pharmacologic treatment, outcomes in the TAU alone group were assumed to be relatively stable over the 3-year time horizon with 80% of TAU alone patients remaining in their outcome category at enrollment.

### Program Attrition

Attrition was considered in the models, including from the DTx and the health plan overall [24]. DTx attrition was considered at several time points. Since long-term attrition rates are not known for DTx, attrition rates were modeled after those commonly seen with pharmacotherapy in HTN and T2DM [25-28]. During year 1, patients were classified as terminating if they did not engage with the app, if they withdrew or clinical outcomes did not improve after 3 months (20% attrition), or if clinical improvements were not durable at year end (additional 20% attrition). Years 2 and 3 also included the withdrawal or lack of durable clinical responses as attrition factors (additional 10% each year). If patients attrited in year 1, their clinical outcomes returned to their enrollment values. During years 2 and 3, program withdrawals were considered to return to average TAU only outcomes for that year. Note that proportions of patients listed as achieving given clinical outcomes at the end of each year (Figure 1 and Multimedia Appendix 1, Figures S1 and S2) are for patients who remained with the DTx + TAU cohort.

### Clinical Effectiveness and Clinical Inertia

Model inputs regarding DTx clinical effectiveness are summarized in Table 1. By the end of year 1, the mean change for enrollees remaining in the program was –0.8% (HbA_1c_) in the T2DM patient population [12] and –11 mm Hg (SBP) in the HTN population [13]. For patients remaining in the DTx program, small improvements were assumed for years 2 and 3 in the base case. Complete descriptions of the 3-year decision trees are provided in Multimedia Appendix 1 (Figures S1 and S2 and Tables S1 and S3).

Benefits from improved clinical outcomes are not assumed to be instantaneous. The base case assumes that for active patients, there were delays of 6 months before medications were reduced based on sustained outcomes and 3 months for any reduction in cardiovascular disease (CVD) hospitalization risk. While we do consider CVD-related hospitalizations, the current evidence base on DTxs does not support accounting for any potential differences in CVD-related mortality. In addition, while many patients in category 2 would be candidates for medication deprescription, we included a parameter controlling the portion of patients in category 2 managed using DTxs alone, without pharmacologic treatment.

**Table 1:** Model input parameters and ranges for sensitivity analysis.

Parameter		T2DM^a^			HTN^b^	
		Base	Range for SA^c^	Base		Range for SA
Age in years, mean		50	±5	50		±5
Enrolled in category 1, (%)		0	—^d^	0		—
Enrolled in category 2, (%)		0.47	—	0.37		—
Enrolled in category 3, (%)		0.34	—	0.19		—
Enrolled in category 4, (%)		0.19	—	0.44		—
**Comorbid conditions, (%)**						
	T2DM	—	—	33		±20
	HC^e^	60	+10/–30	—		—
	HTN	60	+10/–30	—		—
**Digital therapeutic performance**						
	Patients improving ≥1 category from baseline, %	62	+10/–33	87		+10/–33
	Mean improvement by end of year 1	0.8^f^	+20/–40	11^g^		+20/–40
**Medications and resource use**						
	Category 2 pts not on medications, %	25	0/50	25		0/50
	T2DM medications: annual cost ($), range (%)	2466	±20	—		—
	T2DM medications: HbA_1c_^h^ gradient for use, slope	0/0.33/1.2/2.2	±10	—		—
	HC meds: annual cost ($), range (%)	775	±20	—		—
	HC meds: lipid gradient for use, slope	0.5/0.8/1.5/2	±10	—		—
	HTN meds: annual cost ($), range (%)	1557	±20	—		—
	HTN meds: SBP^i^ gradient for use, slope	0/0.15/0.9/1.8	±10	—		—
	CVD^j^ event cost ($), range (%)	116,423	±20	—		—
	HRs^k^ of CVD rate by HbA_1c_ level, slope	1/1/1.25/1.98	±10	—		—
**Health state utilities (from 0 to 1.0)**						
	T2DM: category 1 (increment)	0.02	±20	—		—
	T2DM: category 2 without medications	0.82	—	—		—
	T2DM: category 2 with medications (increment)	–0.02	±20	—		—
	T2DM: category 3 (increment)	–0.035	±20	—		—
	T2DM: category 4 (increment)	–0.025	±20	—		—
	HTN: category 1 (increment)	—	—	0.025		±20
	HTN: category 2 without medications	—	—	0.83		—
	HTN: category 2 with medications (increment)	—	—	–0.01		±20
	HTN: category 3 (increment)	—	—	–0.03		±20
	HTN: category 4 (increment)	—	—	0		±20
	CVD event (increment)	–0.1	±20	–0.1		±20
**Month in year 1 economic benefits realized**						
	Months required for reduction in medications	6	±3	6		±3
	Months required for CVD risk reduction	3	±1	3		±1
	Discount rate, %	3	0/5	3		0/5

^a^T2DM: type 2 diabetes mellitus.

^b^HTN: hypertension.

^c^SA: sensitivity analysis.

^d^Not applicable.

^e^HC: high cholesterol.

^f^Hemoglobin A_1c_ level.

^g^mm Hg.

^h^HbA_1c_: hemoglobin A_1c_.

^i^SBP: systolic blood pressure.

^j^CVD: cardiovascular disease.

^k^HR: hazard ratio.

### Patient Data

Enrolled patients were assumed to be 50% female with a mean age of 50 years in the base case (Table 1). Detailed clinical characteristics by outcome category for each condition were based on LookAHEAD [11], a large prospective study of conventionally delivered intensive lifestyle intervention in T2DM (Multimedia Appendix 1, Table S4). Table 1 also describes the distribution of enrollees by clinical category for each disease state. For T2DM, these are based on data from large US community practices [23]. Distributions of HTN enrollees by clinical category are based on guidelines [9]. Assumed comorbid prevalences are also included in Table 1 [29,30].

### Resource Use, Costs, and Health State Utilities

Medication and cardiovascular event costs were based on a survey of the recent literature [31-36]. Medication costs for T2DM [32] do not include insulin costs since we assume the majority of enrolled patients are not insulin dependent. Medication costs for hypertension are estimated for a nationally representative hypertensive patient population [35]. All future costs and benefits were discounted at 3% and adjusted to 2018 dollars.

The models attempt to reflect actual clinical practice by varying medication intensity by disease severity. A recent analysis of administrative claims classified T2DM patients into 4 cohorts based on diabetes-related drug utilization [21]. The study found that patients with HbA_1c_ >8.9% had diabetes-related drug costs over 9 times those who recently initiated monotherapy with an average HbA_1c_ of 8.0%. Patients moderately controlled on monotherapy were 1.43 times more costly than diagnosed patients without treatment (mean baseline HbA_1c_ of 6.4%), while those poorly controlled were 2.44 to 2.98 times more costly. An analysis of commercial health plan data examined resource utilization among adult T2DM patients categorized by HbA_1c_ at baseline [37]. With HbA_1c_ <7.0% as the reference, patients with HbA_1c_ levels of 7.0% to 8.0% had 1-year prescription costs 45% higher, those with levels 8.0% to 9.0% were 108% higher, and those with levels >9.0% were 131% higher. Similar relationships between HbA_1c_ and diabetes-related hospitalizations were found in an analysis of 200,000 patients with either type 1 or type 2 diabetes [38].

Relationships between medication intensity and disease severity have not been as thoroughly studied in HTN, although these trends are clearly reflected in treatment guidelines. Medication intensity gradients assumed for HTN are weaker than the literature-based estimate used in the T2DM model. CVD event rates are based on hazard ratios by HbA_1c_ level [22] and by the Framingham 10-Year Risk of General Cardiovascular Disease risk equation for HTN-specific outcomes [39].

Health state utilities were drawn from the literature [40,41]. Baseline utilities are defined for category 2, with increments for improved status or less drug utilization and utility decrements for worse health states (see Multimedia Appendix 1 tables for calculated utilities for all health states in the models).

### Sensitivity Analyses

One-way deterministic sensitivity analyses were performed to assess robustness of the base case evaluation of potential health resource saving. Most parameters varied by ±20% (Table 1) with the primary exception being DTx effectiveness. Since real-world effectiveness of any single intervention may be lower than demonstrated in a controlled small-cohort setting, our sensitivity analyses assume a conservative, asymmetric range of 40% below the base case estimate and only 20% above.

## Results

**Figure 2:**
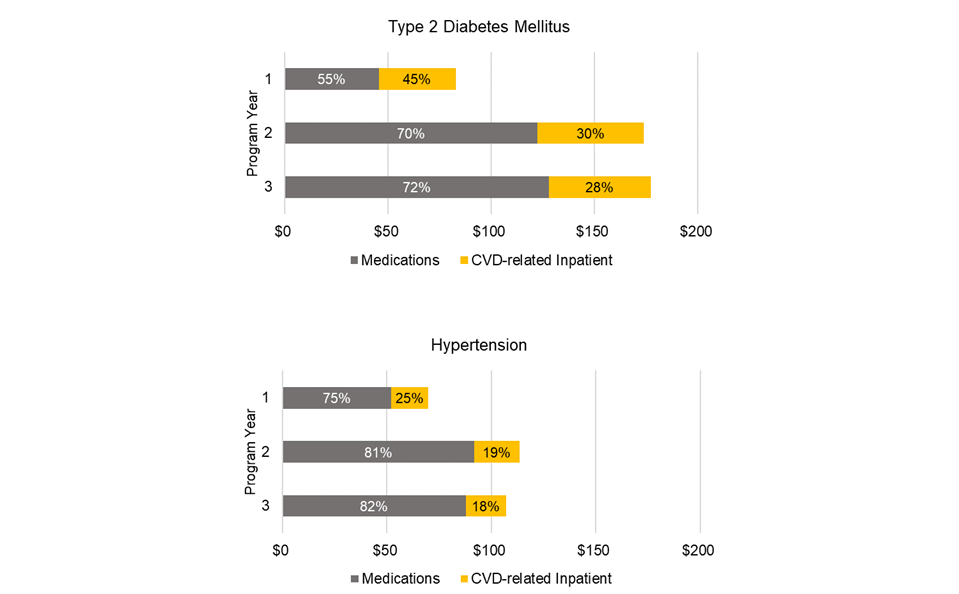
Base case health resource use savings and contributions to estimated savings. Cost estimates are per enrollee per month in year 1 dollars by patient population.

Savings in T2DM are estimated at $83 per participant per month (PPPM) in year 1 and rise to $174 to $178 in years 2 and 3, respectively. Year 1 savings in HTN are estimated at $70 PPPM rising to $113 and $107 in years 2 and 3, respectively. Estimated year 1 savings in HRU (Figure 2) are lower than savings in subsequent years due to delays in realizing economic benefits of reducing medications and CVD-related hospitalizations. The primary driver of these differences across disease states is the magnitude of potential reduction in medication costs, with higher average drug costs in T2DM. In addition, the Framingham equation reflects a less steep CVD risk trend across the outcome categories in HTN compared with the trend between CVD risk and HbA_1c_ [22].

The estimated savings reflect changes in clinical effectiveness over time (Figure 1 and Multimedia Appendix 1, Figures S1 and S2). The largest improvements in outcomes are assumed in year 1, with incremental improvements in years 2 and 3 for DTx + TAU. The most important factor of clinical effectiveness driving estimated economic benefits is the differential between the two cohorts in each patient population (Multimedia Appendix 1, Table S4). The effectiveness of TAU alone is comparable in both patient populations, accounting for differences in severity at enrollment. However, DTx effectiveness in HTN is assumed to be slightly more effective than in T2DM. The majority of estimated HRU savings are due to potential reductions in medication costs for both patient populations and across program years. Medication reductions contribute a larger proportion of savings in years 2 and 3 for T2DM due to higher assumed average medication costs and clinical inertia (Figure 2). Due to the shallow trend in CVD risk across HTN outcome categories, inpatient costs represent a relatively smaller contribution to total savings.

**Figure 3:**
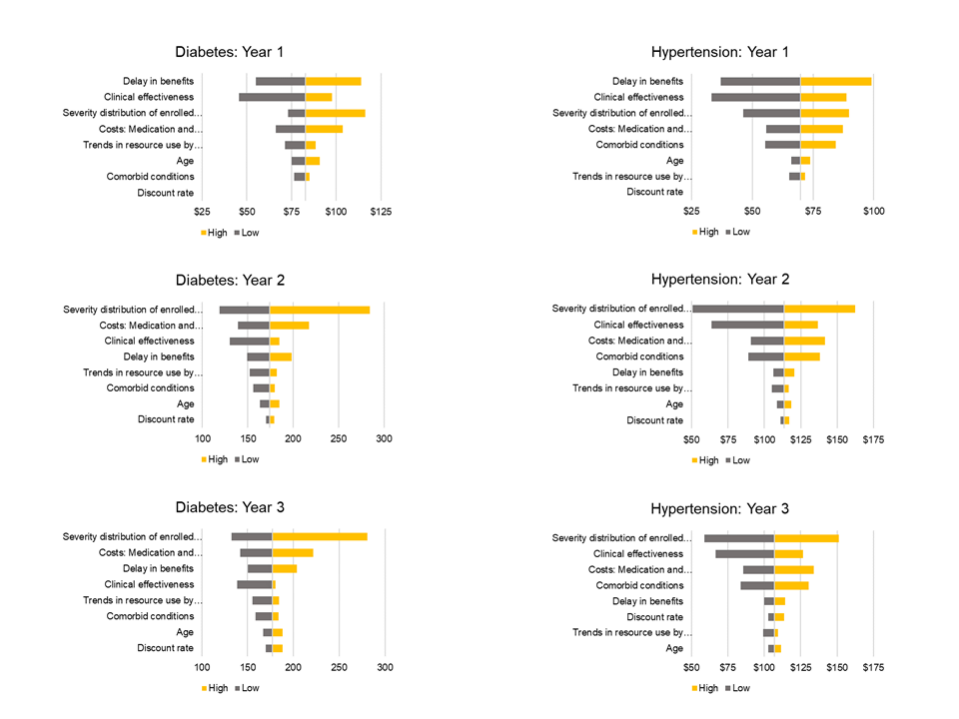
Health resource use sensitivity analysis by patient population.

In the sensitivity analyses (Figure 3), assumptions regarding the distribution of enrollee disease severity are a significant driver of uncertainty for the T2DM population, although this is less important in HTN. In the high-cost, high-effectiveness scenario, only more severe patients (categories 3 and 4) are enrolled (0% category 2). Since the base case assumes nearly half of enrolled T2DM patients are in category 2, estimated savings rise when more patients can experience larger improvements in clinical outcomes. Notably, assumed HRU costs are not the largest driver of uncertainty in year 1, while medication and hospitalization costs are an important driver of uncertainty in subsequent years. Notably, delays in realizing economic benefits of improving HbA_1c_ and SBP levels are a large driver of uncertainty for year 1 and moderately important in years 2 and 3. In T2DM, for example, greater delays in realizing benefits reduces estimated HRU savings to $55 PPPM from the baseline of $83, while shorter delays (3 months vs the baseline 6 months) result in estimated savings of $98 PPPM in year 1. Clinical inertia is a smaller contributor to uncertainty in years 2 and 3 for T2DM, resulting in variances of about 15% for those years. Estimates for HTN indicate that clinical inertia assumptions vary HRU savings by 42% to 47% in year 1. Clinical inertia becomes a small driver of uncertainty in subsequent years in HTN. Assumptions regarding DTx clinical effectiveness (Figure 1 and Multimedia Appendix 1, Figures S1 and S2) are important drivers of uncertainty. However, in the T2DM population, these assumptions are less important than HRU costs, severity distribution, clinical inertia, and comorbidities. Also, with the lower drug costs and weaker trend in CVD risk in HTN, clinical effectiveness is a relatively greater driver in this condition.

**Figure 4:**
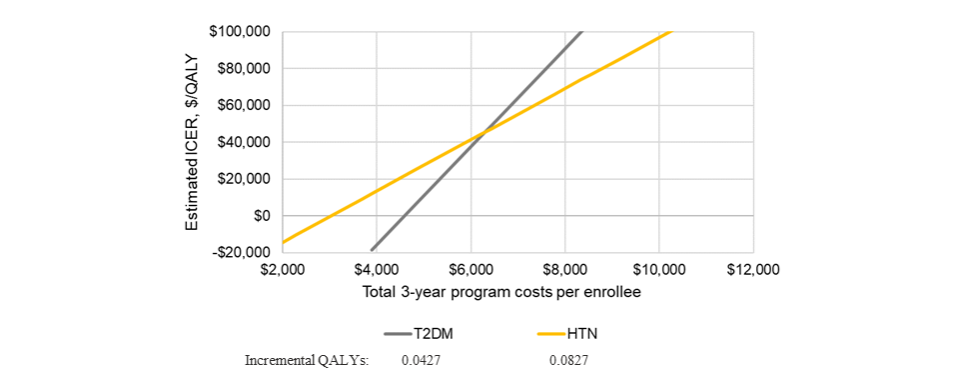
Cost-effectiveness threshold curves by patient population.

The threshold analysis (Figure 4) examines cost effectiveness with varying levels of total DTx program costs over the 3-year time horizon. At a willingness-to-pay threshold of $100,000/quality-adjusted life year (QALY), the DTx + TAU combination is estimated to be cost effective at total 3-year program costs of $8348 (T2DM) and $10,212 (HTN). At threshold of $50,000/QALY, these values are $6468 (T2DM) and $6620 (HTN). These estimates are less elastic for T2DM due to higher drug costs and stronger relationships between outcome categories and CVD hospitalization.

## Discussion

### Principal Findings

The addition of DTxs to conventional pharmacologic treatment as usual in cardiometabolic diseases holds the potential to reduce HRU costs. Sensitivity analyses show that potential HRU savings were sensitive to assumptions regarding the magnitude of HRU costs offset by the DTx, severity distribution of enrolled patients, estimates of DTx clinical effectiveness, and measures of clinical inertia. Cost-effectiveness analyses, limited by the 3-year time horizon, estimated that at a willingness-to-pay threshold of only $50,000/QALY, addition of the DTx would be cost effective at total 3-year DTx intervention costs of up to the average total medication costs for these diseases over the same period.

This study has demonstrated some differential impacts of DTxs in two cardiometabolic diseases and suggests hypotheses for further exploration. One area is the finding that the severity of enrolled patients greatly affects the potential benefits of the DTx. Sensitivity analyses (Figure 3) showed that severity at enrollment was the third largest driver of uncertainty in year 1 and the largest in years 2 and 3. Restricting enrollment to a moderately severe to severe population (categories 3 and 4 only) increased estimated PPPM HRU savings by 40% to 60% in T2DM and 30% to 40% in HTN. As an extension, we also examined the impact (results not shown) of enrolling only the most severe (category 4) patients, which resulted in lower HRU savings in years 2 and 3 relative to enrolling category 3 and 4 patients. This is due to our modeled assumptions that enrolled category 4 patients are more resistant to improvements than are category 3 patients. The impact of severity at enrollment varies across disease states and is due in part to the distribution of HTN versus T2DM severity in a typical commercially insured population. For example, there are far more severe (category 4) enrolled HTN patients than category 4 T2DM patients, and there is a small proportion of moderately severe (category 3) enrolled HTN patients. Estimated gains in both T2DM and HTN are substantial. However, most improvements occur in year 1 for HTN patients with modest improvements in years 2 and 3. Whereas in T2DM, with fewer severe (category 4) patients enrolled, the estimated savings in years 2 and 3 are over twice those estimated in year 1. Future economic evaluations or projections of DTxs should consider the effect of baseline disease severity on the cost benefits of the treatment.

Another hypothesis relates to how clinical inertia could limit the economic benefits realized from digitally delivered behavioral interventions that treat cardiometabolic diseases. As an example, participants who experience a sustained improvement in HbA_1c_ below 6.5% should be considered for medication reduction. Research is needed to quantify the extent of clinical inertia observed in real-world implementations of DTxs. In addition, apps that work with DTxs, such as clinical decision support software, should be explored as solutions to clinical inertia.

### Limitations

Our analysis has several limitations. First, our results are based on simple decision analytic models that rely on estimates of clinical effectiveness drawn from limited cohort studies with DTxs. T2DM and HTN are complex, chronic conditions, and sophisticated techniques coupled with detailed effectiveness data are required to accurately simulate treatment outcomes over a 10- to 20-year time period. However, over the short time horizon appropriate in this setting, our approach likely provides valid directional estimates of potential benefits to US commercial payers. In addition, since patient-level benefits will likely continue after payer reimbursements end, the relatively short time horizon will generate conservative estimates of cost effectiveness. While the potential improvements in clinical outcomes for patients responding to DTxs are consistent with prior experience (eg, an average 0.8% reduction in HbA_1c_ for T2DM patients at 13 weeks), sensitivity analyses confirmed that these are important drivers of potential savings. Performance of DTxs in real-world settings is required to further validate their potential for cost savings. An important driver of real-world effectiveness is program attrition. While we account for attrition throughout the program, with 36% of enrollees withdrawing by the end of year 1 alone, attrition in actual practice may be higher, reducing the total economic savings possible. Second, the analyses assume that the DTx is the only intervention alongside treatment as usual. This is relatively common in economic evaluations of digital health [18]. However, in practice, particularly in US commercial health plans, patients may participate in multiple cardiometabolic disease management programs. In such situations, it will be difficult to associate the specific impact of one intervention versus another, and the net result is uncertain a priori. To the extent that there is overlap in content of a DTx and conventional interventions, the improvements attributable solely to the DTx may be less than estimated in our modeled scenarios. Conversely, there may be synergistic effects between DTxs and conventional interventions, with larger net benefits. More research is required on the best ways to implement DTxs, including analyses of real-world observational data of DTxs using econometric or machine learning methods to distinguish individual effects of multiple interventions. Additionally, no costs of adverse events were included in these analyses. For patients with the same clinical outcome category receiving similar medication regimens, the medication-related adverse events and associated costs would be comparable across DTx + TAU versus TAU alone cohorts. However, no known clinical adverse events are associated with the DTx, and a larger proportion of patients will be managed to improved outcomes not requiring pharmacologic treatment while using the DTx. Thus, not including adverse events may bias the estimates of DTx benefits downward slightly. Finally, while we account for attrition from the DTx cohort, we don’t directly take treatment adherence to TAU into account. However, our drug cost estimates are drawn from published analyses of commercial claims data for diagnosed patients, which account for some level of nonadherence in a typical health plan.

### Comparison With Prior Work

Averaged over the 3-year time horizon, estimated savings were $145 PPPM for the T2DM population and $97 PPPM in HTN patients. Given the drug costs for T2DM and HTN drawn from the peer-reviewed literature, these savings represent approximately 22% to 29% of the total estimated medical costs for an average patient treated as usual. This estimate is in line with the findings of the economics benefits of a mobile DTx for heart failure patients, which found a 33% reduction in total management and treatment costs, and an app-based glucose monitoring program, which found a 22% reduction in total medical spending [19,42].

### Conclusions

DTXs for T2DM and HTN patients may provide substantial improvements in patient outcomes resulting in lower HRU and costs when compared with standard pharmacologic-based treatment as usual. Clinical inertia may be a barrier to realizing the benefits of DTxs.

## Appendix

Multimedia Appendix 1 Supplementary figures and tables.

## Abbreviations

CHEERS: Consolidated Health Economic Evaluation Reporting Standards
CVD: cardiovascular disease
DTx: digital therapeutic
HbA_1c_: hemoglobin A_1c_
HRU: health resource utilization
HTN: hypertension
PPPM: per participant per month
QALY: quality-adjusted life year
SBP: systolic blood pressure
T2DM: type 2 diabetes mellitus
TAU: treatment as usual
