# Supplementary material for: Estimating the Impact of Novel Digital Therapeutics in Type 2 Diabetes and Hypertension: Health Economic Analysis
Source: J Med Internet Res. 2019 Oct 9;21(10):e15814. doi: 10.2196/15814 (PMC6914106; doi:10.2196/15814)
Supplement: Multimedia Appendix 1 [file jmir_v21i10e15814_app1.pdf]

Multimedia Appendix 1. Supplementary figures and tables.

Figure S1. Complete 3-year patient flow diagram for type 2 diabetes mellitus

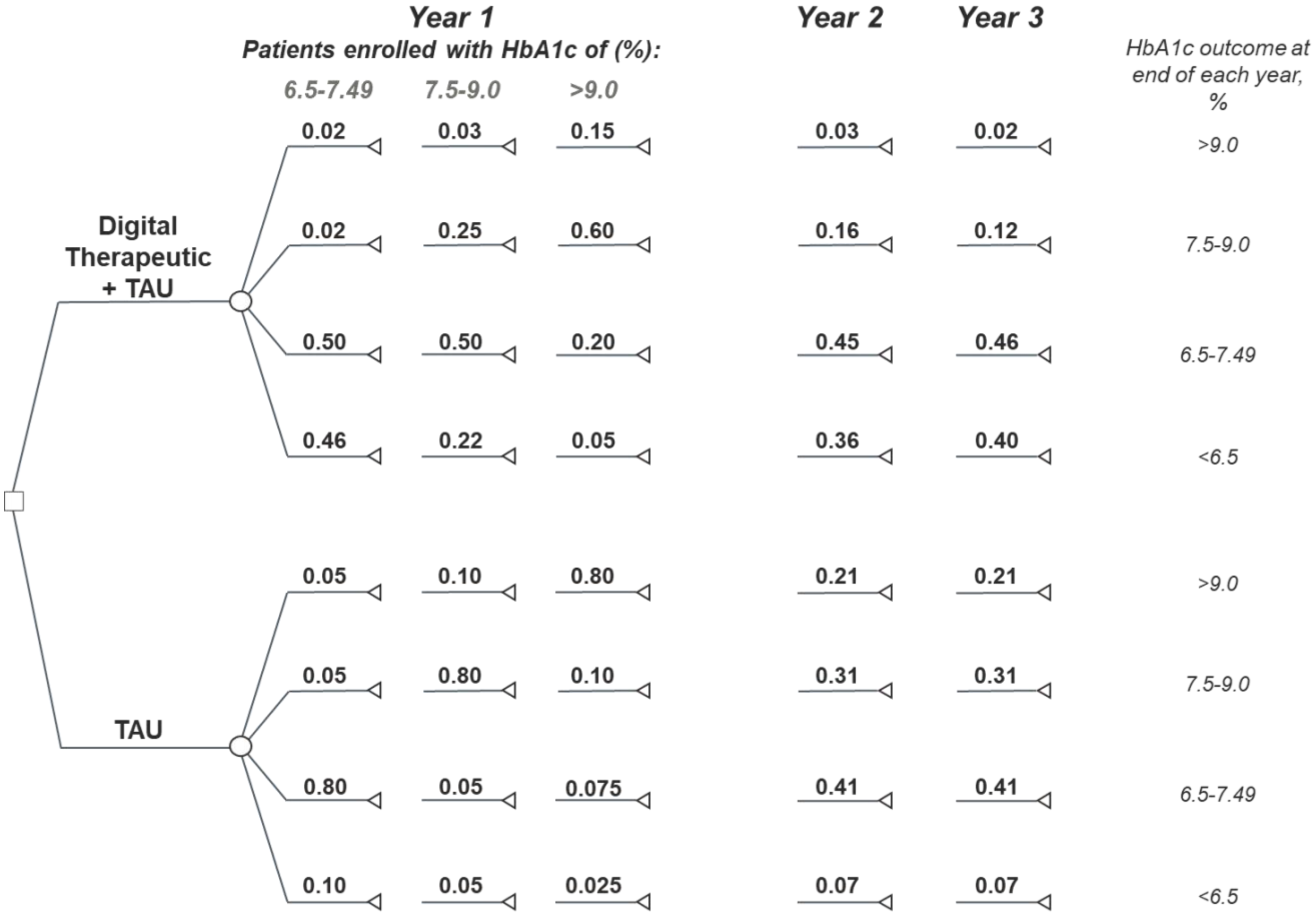

Note: DTx+TAU outcomes apply only for those patients who remain in the DTx program, see discussion on attrition in Methods section

Table S1. Proportion of remaining patients by outcome category and time point, Type 2 diabetes mellitus

HbA1c Level, %	Enrollment	EOY1	EOY2	EOY3
Digital Therapeutic + TAU				
>9	0.19	0.05	0.03	0.02
7.5-9	0.34	0.21	0.16	0.12
6.5-7.5	0.47	0.44	0.45	0.46
<6.5	0	0.30	0.36	0.40
TAU Alone				
>9	0.19	0.21	0.21	0.21
7.5-9	0.34	0.31	0.31	0.31
6.5-7.5	0.47	0.41	0.41	0.41
<6.5	0	0.07	0.07	0.07

Figure S2. Complete 3-year patient flow diagram for hypertension

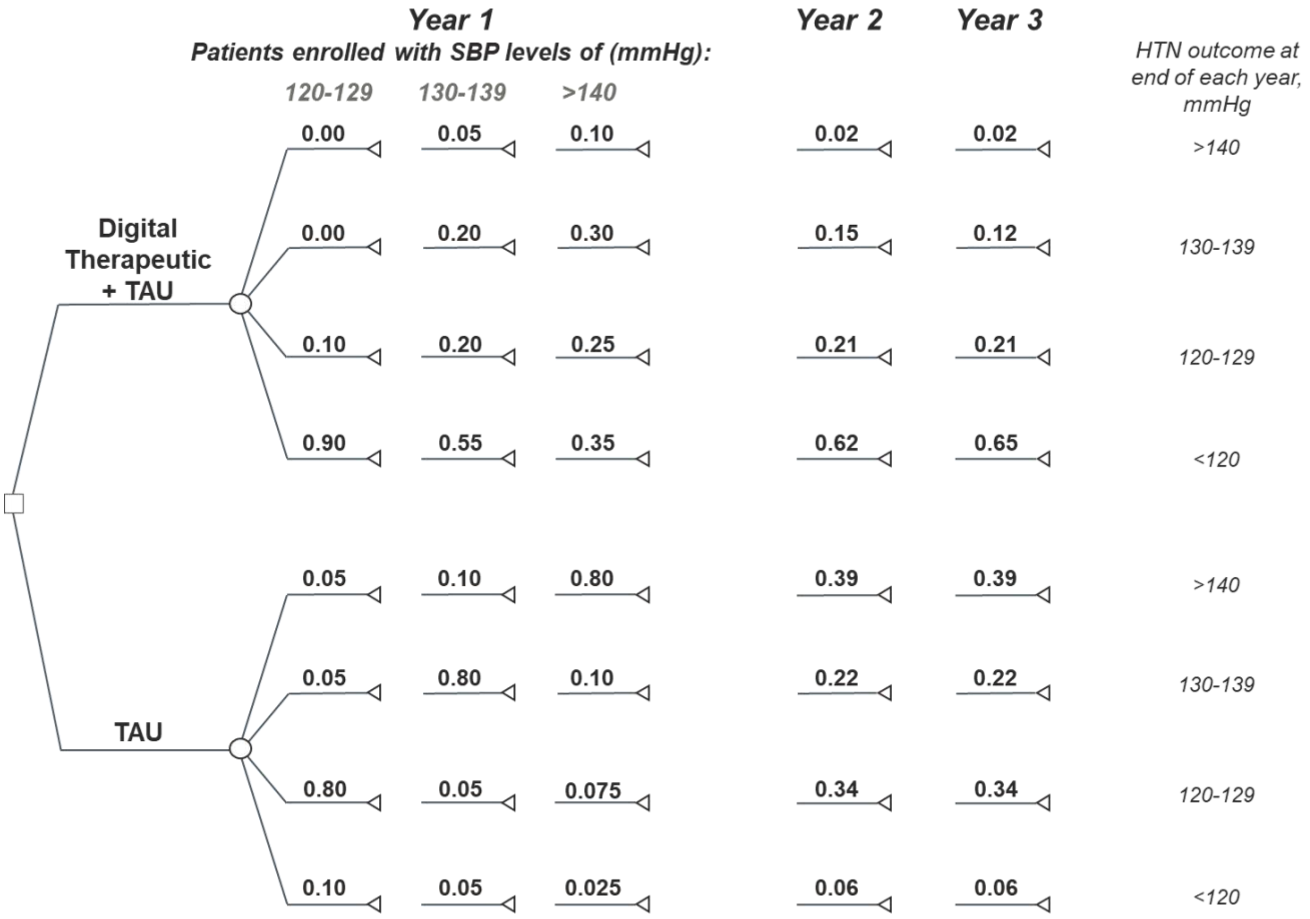

Note: DTx+TAU outcomes apply only for those patients who remain in the DTx program, see discussion on attrition in Methods section

Table S2. Proportion of remaining patients by outcome category and time point, Hypertension

SBP Level, mmHg	Enrollment	EOY1	EOY2	EOY3
**Digital Therapeutic + TAU**				
**≥140**	0.44	0.05	0.02	0.02
**130-139**	0.19	0.17	0.15	0.12
**120-129**	0.37	0.18	0.21	0.21
**<120**	0	0.59	0.62	0.65
**TAU Alone**				
**≥140**	0.44	0.39	0.39	0.39
**130-139**	0.19	0.22	0.22	0.22
**120-129**	0.37	0.34	0.34	0.34
**<120**	0	0.06	0.06	0.06

Table S3. Clinical parameters used in Framingham equations for CVD risk[a]

	T2DM model outcome categories- HbA1c Levels, %			
**Mean value**	**<6.5**	**6.5-7.49**	**7.5-9%**	**>9%**
SBP, mmHg	117.0	125.3	139.0	158.2
HDL, mm/dL	48.7	44.8	38.2	29.0
LDL. mg/dL	105.4	110.6	119.3	131.4
Triglyceride value	137.3	171.6	228.8	308.9
Total Cholesterol	181.6	189.8	203.3	222.2
	**HTN model outcome categories– SBP levels, mmHg**			
**Mean value**	**<120**	**120-129**	**130-139**	**>140**
SBP, mmHg	115.0	125.0	135.0	145.0
HDL, mm/dL	49.7	44.9	40.1	35.3
LDL. mg/dL	104.2	110.5	116.8	123.1
Triglyceride value	128.9	170.6	212.2	253.9
Total Cholesterol	179.6	189.5	199.4	209.2

[a] interpolated from LookAhead [11]

Table S4. Calculated proportions of non-attriting patients improving ≥1 category, %

Year	T2DM		HTN	
	Digital therapeutic + TAU	TAU Alone	Digital therapeutic + TAU	TAU Alone
**1**	62	12	87	14
**2**	69	12	92	14
**3**	72	12	94	14

Table S5. Calculated health state utilities for the included clinical outcome categories

Clinical Outcome Category	T2DM	HTN
**1**	0.8393	0.8544
**2 without medication**	0.8187	0.8292
**2 with medication**	0.7987	0.8192
**3**	0.7633	0.7889
**4**	0.7374	0.7885
